# Intrinsic capacity and the physical frailty phenotype as complementary predictors of mortality in older Indian adults

**DOI:** 10.21203/rs.3.rs-10218104/v1

**Published:** 2026-07-13

**Authors:** Bhrigu Jain, Aparajit Dey, Sharmistha Dey

**Affiliations:** 1 Department of Medicine and Geriatrics, Medanta — The Medicity, Gurugram, Haryana, India.; 2 Department of Geriatric Medicine, Artemis Hospitals, Gurugram, India.; 3 Department of Biophysics, All India Institute of Medical Sciences, New Delhi, India.

**Keywords:** intrinsic capacity, frailty, physical frailty phenotype, cognition, mortality, India

## Abstract

**Purpose.:**

Intrinsic capacity (IC) and frailty both predict mortality and are increasingly advocated for organising older people’s care, yet whether they are distinct or redundant is unclear. We examined whether intrinsic capacity adds to the physical frailty phenotype in predicting mortality, which component carries that information, and whether the physical phenotype alone leaves a high-risk group unidentified.

**Methods.:**

We studied 3,260 adults aged ≥60 in the Longitudinal Aging Study in India – Diagnostic Assessment of Dementia (LASI-DAD), followed up to seven years (642 deaths). Intrinsic capacity (six domains) and the Fried phenotype were entered into Cox proportional-hazards models; IC was decomposed by domain, discordant participants examined, and findings tested in split-sample analysis.

**Results.:**

The two measures were only moderately correlated (r = −0.35) and each predicted mortality independently (adjusted HR 0.85 and 1.26 per SD), indicating complementarity rather than redundancy. The only domain adding information beyond the physical phenotype was cognition (HR 0.73 per SD; likelihood-ratio χ^2^ = 53, p < 0.001) — the one domain a physical assessment cannot capture. Physically robust but low-IC participants died at roughly twice the rate of robust, high-IC participants (HR 2.0), comparable to those meeting frailty criteria, with the excess attributable to cognition. Findings replicated in both sample halves.

**Conclusion.:**

In older Indians, intrinsic capacity and the physical phenotype capture different vulnerabilities; the information the physical phenotype lacks is specifically cognitive. A physically robust older adult with low IC may carry mortality risk comparable to a frail individual while undetected by physical frailty screening.

## 1. Introduction

Frailty has become one of the organising concepts of geriatric medicine: a state of diminished physiological reserve in which a minor insult — an infection, a new medication, a fall — can precipitate disproportionate decline in an older person [1,2]. Two approaches to measuring it dominate clinical practice. Fried’s physical phenotype defines frailty as a syndrome of five observable signs — weak grip, slow gait, exhaustion, weight loss, and low activity — grounded in a self-reinforcing cycle of sarcopenia and energy depletion [3]. The deficit-accumulation index takes the opposite approach, counting health problems across diseases, symptoms, disabilities, and abnormal laboratory results, on the principle that the more deficits a person accumulates, the frailer they are [4]. Both predict death, disability, and hospitalisation, and both are embedded in clinical guidelines [2,5]. They are not the same instrument, and they do not measure the same thing.

The World Health Organization has advanced a complementary perspective on healthy ageing: intrinsic capacity (IC), the composite of all the physical and mental abilities a person can still draw upon [6,7]. Where frailty describes what has been lost, intrinsic capacity describes what remains. It is the centrepiece of the WHO’s healthy-ageing framework and of ICOPE, its programme for community-based assessment of older adults, and it is explicitly multidomain — cognition, psychological state, vitality, locomotion, vision, and hearing — designed to be assessed even in primary care in lower-resource settings [8,9,10].

That intrinsic capacity predicts survival is well established. A meta-analysis of 37 studies and more than 200,000 older adults found higher IC strongly associated with lower mortality (pooled hazard ratio 0.57) [11]. The association holds across diverse populations: in the 10/66 cohorts spanning Latin America, India, and China, impairment in IC domains predicted both death and incident dependence [12], and in long-running cohorts elsewhere IC has tracked mortality over one to two decades [13]. The LASI-DAD cohort studied here has likewise reported lower mortality with higher IC, with cognition, nutrition, and locomotion the strongest domains [14]. We therefore take this association as established and do not revisit it.

The open question is a different one: how intrinsic capacity relates to frailty. The two have been described as “distinct but related,” lying along a shared continuum in which advanced frailty corresponds to a profound loss of IC [15], and a recent WHO-led synthesis has reframed them as complementary rather than competing [16]. Empirical work has begun to separate them, identifying overlapping but distinct constructs [17,18]. This framing, however, obscures an important point: “frailty” is not a single entity. A deficit index — broad, and built in part from the same functional, cognitive, and mood items that constitute IC — must overlap with it; the physical phenotype, narrow and bodily, need not. Whether IC is redundant or complementary therefore depends on which frailty measure is used, and the cleaner, more informative comparison is with the physical phenotype, the instrument least likely to overlap with intrinsic capacity. The practical question becomes: once the physical phenotype has been assessed, does intrinsic capacity add anything to the prediction of death, and if so, which component?

There is a specific reason to expect that the answer is cognition. The physical phenotype, by construction, measures physical performance; it contains no assessment of memory or thinking. If the value IC adds over the physical phenotype were to arise from its cognitive domain, then a physical frailty screen, however well performed, would be structurally blind to a class of vulnerable older adults. That cognition and physical frailty together identify particularly high-risk individuals is itself well recognised: it is the basis of “cognitive frailty,” defined by consensus as co-existing physical frailty and cognitive impairment without dementia [19,20], and a 17-country analysis recently placed the mortality of cognitive frailty (subhazard ratio 2.34) well above that of either component alone [21,22]. Cognitive impairment short of dementia has similarly been shown to add to physical frailty in predicting death and disability [23]. Almost all of this work, however, concerns individuals in whom the two conditions coincide. The outcome of those in whom they diverge, physically robust but cognitively impaired, remains unsettled: some cohorts report excess mortality in such individuals [24], whereas others find that the risk attenuates once frailty is accounted for. Moreover, the few studies that have cross-classified older adults by IC and frailty examined self-rated health or short-term disability rather than death [25,26], leaving the question unresolved.

This cohort offered an opportunity to address the question directly. LASI-DAD is a large, population-based study of older Indians with detailed cognitive assessment linked to mortality follow-up. Using it, we asked three questions. First, are intrinsic capacity and the physical phenotype complementary predictors of mortality, or does one render the other redundant? Second, if IC adds predictive information, which of its six domains is responsible? Third, does reliance on the physical phenotype alone leave a recognisable, high-risk group of older adults unidentified?

## 2. Methods

### 2.1 Design and population

We analysed the Longitudinal Aging Study in India – Diagnostic Assessment of Dementia (LASI-DAD), the dementia sub-study of the nationally representative Longitudinal Aging Study in India (LASI) [27,28]. LASI-DAD enrolled adults aged 60 years and over and assessed them with an extensive battery of cognitive tests modelled on the Harmonized Cognitive Assessment Protocol, together with physical performance measures, anthropometry, and informant reports. Baseline (wave 1) data were used for the predictors, and all-cause mortality was ascertained through the cohort’s end-of-life follow-up. From the full sample, we included participants with complete data on all six intrinsic-capacity domains, the physical phenotype, and follow-up status, yielding an analytic sample of 3,260 adults with 642 deaths. Reporting follows the STROBE recommendations for cohort studies (Supplementary Checklist S1) [29].

### 2.2 Intrinsic capacity

Intrinsic capacity was operationalised across its six standard domains — cognition, psychological, vitality, locomotion, vision, and hearing — following the approach used in the companion analysis of this cohort [14], to ensure comparability of the construct (Supplementary Table S2). Cognition was derived from the general cognitive score; the psychological domain from depressive symptoms (reverse-scored so that higher values indicated better state); vitality from nutritional status; and locomotion from a combination of mobility and basic physical function. The two sensory domains were derived from self-reported vision and hearing. Each domain was standardised (z-scored) and oriented so that higher values indicated better IC, and the six were combined into a single standardised composite. Hazard ratios for IC are expressed per standard deviation of this composite.

### 2.3 Physical frailty phenotype

The Fried phenotype was constructed from its five criteria — weakness (grip strength), slowness (gait speed), self-reported exhaustion, unintentional weight loss, and low physical activity — using the cohort’s measured and self-reported items [3]. Participants were scored 0–5 and classified as robust (0 criteria), pre-frail (1–2), or frail (≥3). In the models, the physical phenotype was entered as a continuous score — the number of criteria met, from 0 to 5 — with hazard ratios expressed per standard deviation to match the IC scale.

### 2.4 Outcome

The outcome was all-cause mortality. Follow-up time was measured in years, from baseline assessment to death or to the most recent confirmation of survival.

### 2.5 Covariates

All models were adjusted for age (in years) and sex. The primary models were kept deliberately parsimonious, as the question concerned the relationship between two summary measures of ageing rather than the effect of any individual risk factor; models with fuller covariate adjustment are reported as sensitivity analyses (Supplementary Table S5).

### 2.6 Statistical analysis

The cohort was first described by vital status (Table 1), and the relationships among the measures were examined using Pearson correlations.

**Fig. 1:**
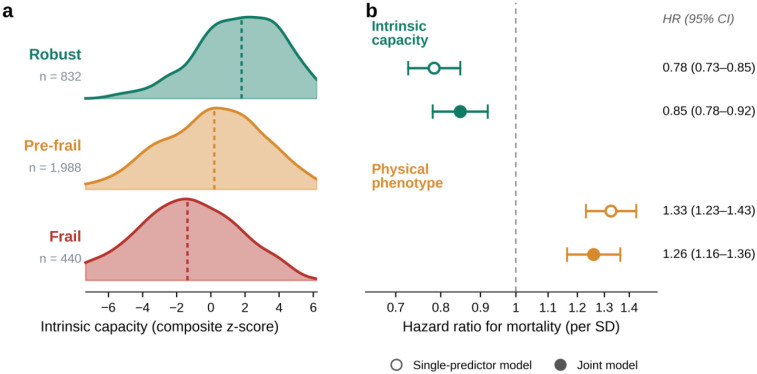
Intrinsic capacity and the physical frailty phenotype are related but distinct, and both predict mortality. (**a**) Distribution (kernel density) of intrinsic capacity, expressed as a composite z-score, within each physical phenotype category; the dashed line marks each group median. IC declines from robust to frail, but the distributions overlap substantially, indicating that the two measures are related rather than interchangeable (IC–physical phenotype correlation r = −0.35). (**b**) Hazard ratios for all-cause mortality (per standard deviation, adjusted for age and sex) for intrinsic capacity and the physical phenotype, each estimated alone (open circles, single-predictor model) and together (filled circles, joint model). Both associations persist with only modest attenuation in the joint model, indicating that the two measures are complementary rather than redundant. CI, confidence interval; HR, hazard ratio; SD, standard deviation

Cox proportional-hazards models were then fitted for mortality. To determine whether IC and the physical phenotype are redundant or complementary, each was examined in a single-predictor model and then in a joint model containing both: attenuation of one association to non-significance would indicate redundancy, whereas persistence of both would indicate complementarity (Fig. 1, Table 2).

**Fig. 2:**
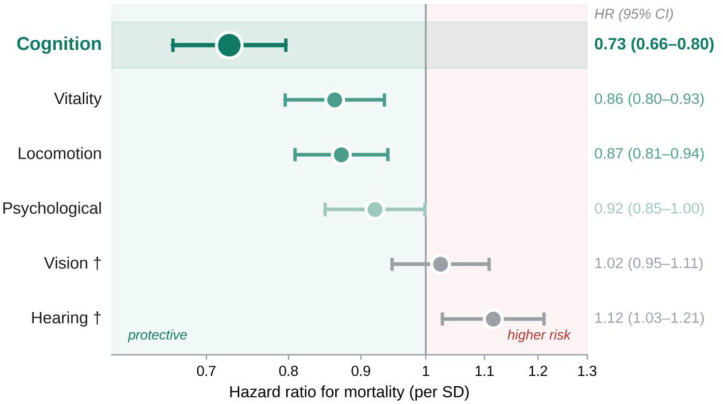
Cognition is the intrinsic-capacity domain that adds prognostic information beyond the physical phenotype. Hazard ratios for all-cause mortality (per standard deviation, each adjusted for the physical phenotype, age and sex) for the six intrinsic-capacity domains, ordered by effect size. Cognition shows the strongest independent association (highlighted) and is the one domain a physical assessment cannot capture; shading indicates the protective (left of 1.0) and higher-risk (right of 1.0) sides of the null. † The two sensory domains performed poorly and were not interpreted; the hearing association ran counter to the expected direction (see Methods and Limitations). HR, hazard ratio; SD, standard deviation

To identify the source of IC’s added predictive value, IC was decomposed by domain. Each of the six domains was fitted alongside the physical phenotype (with age and sex), and then all six domains were entered together with the physical phenotype, to determine which domain retained an independent association once the physical phenotype and the other domains were accounted for (Fig. 2, Table 2). For the leading domain, its contribution beyond the physical phenotype was formally tested with a likelihood-ratio test comparing models with and without it, and the corresponding change in discrimination was quantified (Harrell’s C). The reverse test, the physical phenotype’s contribution beyond cognition, was performed to confirm that complementarity operated in both directions.

**Fig. 3:**
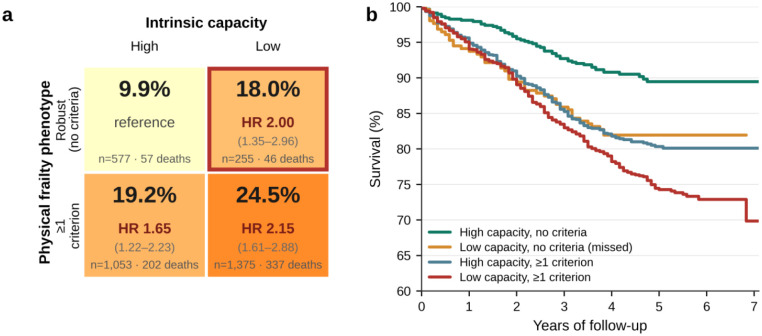
A high-risk group missed by physical frailty screening. (**a**) Age- and sex-adjusted mortality for four groups defined by intrinsic capacity (above or below the cohort median) and the physical phenotype (robust, defined as no criteria, versus one or more criteria). Each cell shows the proportion who died over follow-up, the hazard ratio relative to the robust/high-IC reference group, and the number of participants and deaths. The crimson-bordered cell — participants who are physically robust but have low IC — is the group overlooked by a physical screen; its mortality (18.0%) approaches that of participants meeting one or more physical phenotype criteria. (**b**) Kaplan–Meier survival curves for the same four groups. The low-IC robust group separates early from the reference group and tracks the groups with physical phenotype criteria. HR, hazard ratio

To assess whether a physical frailty screen overlooks at-risk individuals, participants were classified into four groups using two splits. First, the cohort was divided at the median of IC into higher- and lower-IC halves. Second, physically robust participants (those with no frailty criteria) were separated from those with one or more. The mortality of each group was then compared with that of the healthiest group (higher IC and robust), adjusted for age and sex (Fig. 3). Because dichotomising the physical phenotype as “robust versus any criteria” is a coarse division, the principal finding was confirmed by a threshold-independent analysis: among participants with no frailty criteria, we tested whether lower IC and lower cognition specifically remained associated with mortality.

The robustness of the main results was assessed by repeating the principal analyses in two random halves of the sample (split-sample validation), and in sensitivity analyses substituting a deficit-accumulation frailty index for the physical phenotype, applying alternative cut-points, and using fuller covariate adjustment (Supplementary Table S3–5). The proportional-hazards assumption was assessed using Schoenfeld residuals; mild departures were detected for age, intrinsic capacity and the physical phenotype (Supplementary Table S6), as is common with large event counts, and hazard ratios are therefore interpreted as effects averaged over follow-up. Analyses were conducted in Python 3 (pandas, NumPy, SciPy, scikit-learn). A two-sided p value below 0.05 was considered statistically significant.

During the preparation of this manuscript, the authors used a large language model (Claude; Anthropic) to assist with language editing, and formatting. All study design, analysis, and interpretation were performed and verified by the authors, who reviewed and approved the final text and take full responsibility for its content.

### 2.7 Ethics

LASI-DAD received ethical approval from the Indian Council of Medical Research (ICMR) (Ref: 2202-16741/F1) and all collaborating institutions; all participants (or their proxies) provided informed consent. This secondary analysis used de-identified data.

## 3. Results

### 3.1 Cohort

The 3,260 participants had a mean age of 69.2 years (SD 7.1), and 53.3% were women (Table 1). Over a median follow-up of 4.4 years (IQR 3.9–5.3; maximum 7.1), 642 participants (19.7%) died. Decedents were older (73.0 vs 68.2 years), more often men, and had lower body-mass index, fewer years of education, and lower rates of current employment than survivors (all p < 0.001). They were more frequently physically frail (28.0% vs 9.9%) and had lower intrinsic IC and lower cognition (all p < 0.001). Comorbidity burden did not differ between groups (mean count 0.98 vs 0.90; p = 0.10), and the prevalence of hypertension, arthritis, and chronic lung disease was similar by vital status (Table 1).

### 3.2 Intrinsic capacity and the physical phenotype as predictors of mortality

Intrinsic capacity and the physical phenotype were moderately correlated (r = −0.35). IC decreased across physical phenotype categories, with substantial overlap of the IC distributions in the robust, pre-frail, and frail groups (Fig. 1a).

In single-predictor models adjusted for age and sex, both measures were associated with mortality (IC HR 0.78 per SD, 95% CI 0.73–0.85; physical phenotype HR 1.33 per SD, 1.23–1.43). In a joint model containing both, each association persisted with modest attenuation (IC HR 0.85, 0.78–0.92; physical phenotype HR 1.26, 1.16–1.36; Fig. 1b, Table 2), indicating independent associations with mortality.

### 3.3 Domain-specific contribution of intrinsic capacity

Each intrinsic-capacity domain was entered alongside the physical phenotype, age, and sex (Fig. 2, Table 2). Cognition showed the strongest independent association with mortality (HR 0.73 per SD, 0.66–0.80), followed by vitality (0.86, 0.80–0.93) and locomotion (0.87, 0.81–0.94); the psychological domain was borderline (0.92, 0.85–1.00). The two sensory domains were not informative (vision HR 1.02, 0.95–1.11; hearing HR 1.12, 1.03–1.21, in the unexpected direction; see Limitations). In a model containing all six domains and the physical phenotype, cognition retained its association (0.74, 0.68–0.82) while the remaining domains were attenuated or null; the physical phenotype remained associated with mortality (HR ≈ 1.22).

Adding cognition to a model containing the physical phenotype, age, and sex improved model fit (likelihood-ratio χ^2^(1) = 53.4, p = 2.7 × 10^−13^). The physical phenotype likewise improved fit when added to a model containing cognition (χ^2^(1) = 42.1, p < 10^−10^). Discrimination increased marginally: Harrell's C was 0.662 for age and sex, 0.684 with the physical phenotype added, and 0.688 with both IC and the physical phenotype, with the increment attributable to cognition approximately 0.013 (Table 2).

### 3.4 Discordance between IC and the physical phenotype

Participants were cross-classified by IC (above or below the cohort median) and the physical phenotype (robust, defined as no criteria, vs one or more criteria), and mortality was estimated relative to the robust/high-IC group, adjusted for age and sex (Fig. 3a). Among robust participants, those with low IC had higher mortality than those with high IC (18.0% vs 9.9%; HR 2.00, 1.35–2.96); this subgroup comprised approximately one-quarter of robust participants. Their mortality was similar to that of participants with one or more physical phenotype criteria and high IC (19.2%; HR 1.65, 1.22–2.23). Kaplan–Meier curves showed early separation of the low-IC robust group from the reference group (Fig. 3b).

Among robust participants analysed without an IC threshold (Table 2), lower IC was associated with higher mortality (HR 0.74 per SD, 0.61–0.89), as was lower cognition (HR 0.72 per SD, 0.58–0.89).

### 3.5 Sensitivity and replication

In two random split-halves of the cohort, IC and the physical phenotype remained jointly associated with mortality (IC HR 0.81 and 0.87; physical phenotype HR 1.19 and 1.34), the low-IC robust subgroup retained an approximately two-fold hazard (HR 1.87 and 2.12), and cognition retained its association among non-frail participants in both halves. Estimates in the smaller cells were less precise but consistent in direction and magnitude (Supplementary Table S3).

Results were unchanged using alternative cut-points for IC and the physical phenotype and fuller covariate adjustment (Supplementary Table S5). When a deficit-accumulation frailty index [30] replaced the physical phenotype, the index and IC were strongly correlated (r = −0.75); in a joint model, IC was no longer associated with mortality (HR 1.03, 0.92–1.15) and added no discrimination beyond the index (Supplementary Table S4).

## 4. Discussion

### 4.1 Principal findings

In this cohort of older Indians, intrinsic capacity and the Fried physical phenotype were complementary predictors of mortality: each retained an independent association when both were modelled together, and neither rendered the other redundant. The IC domain responsible for this complementarity was cognition, the one domain a physical assessment does not capture. This finding had a direct clinical correlate. Participants who were physically robust but had low intrinsic capacity died at approximately twice the rate of their robust, high-IC counterparts, a risk comparable to that of participants meeting physical frailty criteria; cognition accounted for essentially all of this excess. Such individuals would be classified as low-risk by a physical frailty screen applied in isolation. Notably, decedents were not more multimorbid than survivors, and several individual diagnoses did not differ by vital status, consistent with summary measures of IC and frailty conveying prognostic information that a count of diagnoses does not.

### 4.2 Complementarity depends on how frailty is operationalised

These data provide empirical support for a position the field has advanced largely on conceptual grounds — that IC and frailty are complementary rather than competing constructs [15,16] — while specifying an important qualification. Complementarity is not a general property of IC relative to frailty; it depends on how frailty is operationalised. Relative to the physical phenotype, a narrowly defined physical syndrome, IC is genuinely complementary, because it carries non-physical information the physical phenotype does not assess. Relative to a deficit-accumulation index, IC is largely redundant: the index re-measures several of IC’s own domains, and in our sensitivity analysis it absorbed IC entirely. The two frailty instruments therefore stand in different relationships to IC, and the single term “frailty” conflates them. The clinically and statistically informative pairing is intrinsic capacity with the physical phenotype.

### 4.3 Relation to previous direct comparison

To our knowledge, only one previous study has entered intrinsic capacity and the Fried phenotype into the same mortality model. In the Sydney Memory and Ageing Study (n = 400), Numbers and colleagues reported that the physical phenotype’s association with mortality lost significance when IC was added, whereas IC remained predictive, and concluded that IC was the superior measure [31]. Our results are consistent with this observation but support a different interpretation. In the present, larger sample, both measures retained independent associations in the joint model, indicating complementarity rather than the superiority of either; the distinction carries different clinical implications — use of IC in place of the physical phenotype, versus use of both. That study did not identify which IC domain accounted for its predictive advantage, nor did it examine participants in whom IC and the physical phenotype were discordant — the two questions addressed here. The reproduction of its secondary finding in our data, namely that IC added no information beyond a deficit index, suggests that the underlying structure is not specific to a single cohort or setting.

### 4.4 Relation to the intrinsic capacity–mortality literature

These findings extend rather than duplicate the established literature on IC and survival. The association between higher intrinsic capacity and lower mortality is well documented, in meta-analysis [11], in low- and middle-income country (LMIC) cohorts including the 10/66 studies [12], and in a previous analysis of the present cohort that identified cognition among the leading domains [14]. We did not seek to re-establish this association. The novel contributions are the explicit comparison with the physical phenotype and the two questions that follow from it: which component of IC carries the independent risk, and which patients are missed when only the physical phenotype is assessed. The analysis thus addresses the clinical role of intrinsic capacity in settings where a physical frailty instrument is already in use.

### 4.5 Distinction from cognitive frailty

It might be argued that these findings restate the concept of cognitive frailty, the recognised association between coexisting physical frailty and cognitive impairment and adverse outcomes [19,21,23]. The present contribution differs in an important respect. Most cognitive-frailty research has examined the concordant high-risk group, in whom physical frailty and cognitive impairment coexist and adverse outcomes are expected. The unresolved question concerns the discordant groups. This analysis addresses one such group directly, physically robust but cognitively vulnerable participants, and demonstrates that their mortality matches that of participants meeting physical frailty criteria. Operationalising cognitive vulnerability through the validated WHO intrinsic-capacity construct, and quantifying mortality in this discordant group within an LMIC population is where the novelty lies for present analysis.

### 4.6 Mortality in the physically robust but cognitively vulnerable

Whether cognitive impairment in the absence of physical frailty confers excess mortality has been reported inconsistently. Some cohorts demonstrate clear independent risk [24], whereas others find that the apparent risk attenuates after accounting for physical frailty or disability. The present data support an independent association and may help to explain the discrepancy. The most directly comparable studies, which similarly cross-classified older adults by IC and frailty status, used self-rated health or short-term disability as outcomes; at least one reported that the robust, low-IC group did not differ from the healthy reference on self-rated health [25,26]. The same robust, low-IC group that appears no different from healthy peers on self-rated health is, against mortality, clearly at elevated risk. So outcome choice, not the patients, explains the conflicting prior reports.

### 4.7 Possible explanations for the cognitive signal

The basis for the specific predictive value of cognition cannot be determined from these data, but several non-exclusive mechanisms are plausible. Cognition may function as an integrative marker of brain health, registering vascular, metabolic, and neurodegenerative injury before such injury manifests as reduced strength or gait speed; a declining cognitive score may therefore serve as an early indicator of systemic decline. Part of the association probably reflects reverse causation, in that a proportion of robust, low-cognition individuals are in the preclinical phase of dementia, the pathology of which independently increases mortality. Cognition also underlies the self-management required in later life — adherence to medication, recognition of and response to symptoms, and navigation of health services — such that impairment may result in missed treatment and delayed presentation despite preserved physical function. Cognitive function is further associated with social and economic determinants of survival, including educational attainment and social isolation. These mechanisms cannot be distinguished with cross-sectional data; the relevant point is that the physical phenotype, by assessing only physical performance, captures none of them.

### 4.8 Implications for practice

The principal clinical implication is straightforward: physical frailty assessment should be accompanied by at least a brief assessment of cognition. This is consistent with the WHO ICOPE framework, which treats intrinsic capacity as inherently multidomain [8,10]. ICOPE is not yet routinely implemented, however, and in many settings, including India, frailty screening when performed remains predominantly physical. These findings provide an outcome-based, rather than conceptual, rationale for incorporating cognition: an identifiable and substantial group of older adults who would otherwise be reassured are at a risk of death comparable to those with physical frailty, and brief cognitive assessment is what distinguishes them. For low-resource settings, the localisation of the added prognostic value to cognition, rather than to the sensory or psychological domains, indicates where limited assessment time is best directed. These findings do not argue against use of the physical phenotype, which remained independently predictive and captures physical vulnerability that cognition does not, but against reliance on it alone.

### 4.9 Strengths and limitations

The strengths of this study include its size, its setting in a low- and middle-income country where most older adults live and where comparatively little such research has been conducted, a depth of cognitive assessment uncommon in population-based cohorts, the use of two established instruments, and out-of-sample replication.

Several limitations should be noted. IC and the physical phenotype were each assessed at a single time point; the analysis therefore describes associations with subsequent mortality rather than trajectories, and no causal inference is warranted. The data establish that the robust, low-IC group is at elevated risk but not that intervention would modify it, and intrinsic capacity is known to change over time in ways that themselves predict outcomes [32]. The improvement in overall discrimination with the addition of IC was small; the contribution of this study is the localisation of the additional prognostic information and the identification of an at-risk subgroup, not an improvement in predictive performance. The signal was cognition-predominant but not cognition-exclusive, with modest contributions from locomotion and vitality. The two sensory domains performed poorly, with the hearing domain showing an association opposite to that expected; this likely reflects reliance on brief self-report, and these domains were not interpreted, such that the performance of the composite rests principally on its cognitive, nutritional, and mobility components. The cohort is enriched for detailed cognitive phenotyping, which improves measurement precision but may overstate the absolute magnitude of the cognitive effect relative to unselected populations. The proportional-hazards assumption was mildly violated for age, intrinsic capacity and the physical phenotype, the hazard ratios should therefore be read as effects averaged over the approximately seven-year follow-up rather than as constant instantaneous ratios. Finally, residual confounding cannot be excluded, and the limited content shared by IC and the physical phenotype would, if anything, lead to underestimation of their complementarity.

## 5. Conclusion

Intrinsic capacity and the physical frailty phenotype are complementary predictors of mortality in older Indians, and the information the physical phenotype lacks is specifically cognitive. A physically robust older adult with declining intrinsic capacity may carry a mortality risk comparable to that of a frail individual, while remaining undetected by physical frailty screening. Pairing a brief cognitive assessment with frailty screening is a simple, specific measure that would identify these otherwise unrecognised individuals.

## Supplementary Material

Supplementary Files

This is a list of supplementary files associated with this preprint. Click to download.

- SupplementaryICphenotypecognitionLASIDAD.docx

- S1. STROBE checklist.
- **S2. Intrinsic-capacity domain operationalisation** — variables, scoring and orientation for each domain.
- **S3. Split-sample validation** — full model estimates in each random half (joint models, missed-group hazard, cognition among the non-frail).
- **S4. Frailty-index sensitivity analysis** — using the published LASI-DAD deficit-accumulation frailty index [30] in place of the physical phenotype. IC and the index are near-collinear (r = −0.75); in a joint model IC’s association is fully absorbed (HR 1.03, 0.92–1.15) and it adds no discrimination beyond the index, consistent with a deficit index re-measuring IC’s own domains plus comorbidity. This is why the physical phenotype, which is genuinely distinct from IC, is used as the comparator in the main analysis.
- **S5. Alternative cut-points and fuller-covariate models** — robustness of the joint and discordance results.
- S6. Proportional-hazards diagnostics.

## Figures and Tables

**Table 1.** Baseline characteristics of Cohort, by vital status at end of follow-up (n = 3,260)

Characteristic	All (n = 3,260)	Alive (n = 2,618)	Died (n = 642)	P value
**Demographic and social**				
Age, years, mean (SD)	69.2 (7.1)	68.2 (6.4)	73.0 (8.2)	<0.001
Women, n (%)	1,737 (53.3)	55.9	42.5	<0.001
Currently married, %	2,169 (66.5)	68.3	59.5	<0.001
Illiterate, %	1,828 (56.1)	55.1	60.1	0.02
Education, years, mean (SD)	3.9 (4.7)	4.1 (4.7)	3.3 (4.2)	<0.001
Rural residence, %	2,025 (62.1)	61.9	63.1	0.60
Scheduled Caste/Tribe, %	786 (24.1)	23.3	27.3	0.04
Currently working, %	864 (26.5)	28.3	19.2	<0.001
**Lifestyle**				
Ever smoked, %	715 (21.9)	19.9	30.4	<0.001
Ever used alcohol, %	498 (15.3)	14.4	19.0	0.004
**Clinical**				
Body-mass index, mean (SD)	22.6 (5.0)	22.9 (5.0)	21.3 (4.8)	<0.001
Comorbidity count (0–8), mean (SD)	0.91 (1.02)	0.90 (1.00)	0.98 (1.07)	0.10
Hypertension, %	1,252 (38.4)	38.2	39.3	0.65
Diabetes, %	564 (17.3)	16.4	20.9	0.009
Heart disease, %	204 (6.3)	6.0	7.2	0.33
Stroke, %	76 (2.3)	1.9	3.9	0.005
Cancer, %	28 (0.9)	0.7	1.4	0.15
Chronic lung disease, %	96 (2.9)	2.9	3.0	0.99
High cholesterol, %	150 (4.6)	4.9	3.6	0.20
Arthritis, %	605 (18.6)	18.5	18.8	0.88
**Function**				
Any ADL limitation, %	1,640 (50.3)	48.5	57.8	<0.001
Any IADL limitation, %	1,009 (31.0)	28.3	41.7	<0.001
**Physical frailty phenotype, n (%) — p < 0.001**				
Robust (0 criteria)	832 (25.5)	27.8	16.0	
Pre-frail (1–2)	1,988 (61.0)	62.2	55.9	
Frail (≥3)	440 (13.5)	9.9	28.0	
**Intrinsic capacity, domain z-scores, mean (SD)**				
Composite	0.27 (3.05)	0.47 (2.95)	−0.52 (3.29)	<0.001
Cognition	0.06 (0.97)	0.14 (0.96)	−0.26 (0.95)	<0.001
Psychological	0.04 (0.99)	0.08 (0.97)	−0.10 (1.03)	<0.001
Vitality	0.04 (0.98)	0.10 (0.96)	−0.23 (1.02)	<0.001
Locomotion	0.11 (0.85)	0.17 (0.77)	−0.15 (1.08)	<0.001
Vision^a^	0.04 (0.99)	0.06 (0.97)	−0.04 (1.04)	0.04
Hearing^a^	−0.04 (0.98)	−0.09 (0.99)	0.21 (0.92)	<0.001

Follow-up: median 4.4 years (IQR 3.9–5.3), maximum 7.1; 642/3,260 (19.7%) deaths. For binary characteristics the All column shows n (%); Alive and Died columns show the percentage within each group.

* Mann–Whitney U for continuous variables; χ^2^ for categorical. Higher IC/cognition z indicates better function.

a Sensory domains performed poorly and were not interpreted (see Limitations). ADL, activities of daily living; IADL, instrumental activities of daily living.

**Table 2.** Cox proportional-hazards models for all-cause mortality (n = 3,260; 642 deaths), adjusted for age and sex.

Panel A. Intrinsic capacity and the physical phenotype, modelled separately and jointly *(HR per SD)*		
Predictor	Single-predictor model	Joint model
Intrinsic capacity	0.78 (0.73–0.85)	0.85 (0.78–0.92)
Physical frailty phenotype	1.33 (1.23–1.43)	1.26 (1.16–1.36)

Panel B. Intrinsic-capacity domains and mortality *(HR per SD; each model also adjusted for the physical phenotype, age and sex)*		
IC domain	Adjusted for physical phenotype^a^	Mutually adjusted^b^
Cognition	0.73 (0.66–0.80)	0.74 (0.68–0.82)
Vitality	0.86 (0.80–0.93)	0.94 (0.86–1.02)
Locomotion	0.87 (0.81–0.94)	0.90 (0.83–0.98)
Psychological	0.92 (0.85–1.00)	1.00 (0.92–1.09)
Vision^c^	1.02 (0.95–1.11)	1.11 (1.02–1.20)
Hearing^c^	1.12 (1.03–1.21)	1.07 (0.99–1.17)
a Each domain entered in a separate model, together with the physical phenotype, age and sex.		
b All six domains entered simultaneously in a single model, together with the physical phenotype, age and sex. In this model the physical phenotype remained associated with mortality (HR 1.22, 1.13–1.33 per SD).		
c Sensory domains performed poorly and were not interpreted; the hearing association ran counter to the expected direction (see Limitations).		

Panel C. Discrimination *(Harrell’s C)*	
Model	C
Age + sex	0.662
+ Intrinsic capacity	0.673
+ Physical phenotype	0.684
+ Intrinsic capacity + physical phenotype	0.688
Likelihood-ratio test, cognition added to [physical phenotype + age + sex]: χ^2^(1) = 53.4, p = 2.7 × 10^−13^ (ΔC ≈ +0.013). Reverse test, physical phenotype added to [cognition + age + sex]: χ^2^(1) = 42.1, p < 10^−10^.	

Panel D. Within physically robust participants only *(no physical phenotype criteria; n = 832, 103 deaths; HR per SD, adjusted for age and sex)*	
Predictor	HR (95% CI)
Intrinsic capacity	0.74 (0.61–0.89)
Cognition	0.72 (0.58–0.89)
Threshold-independent analysis restricted to participants meeting no physical frailty criteria, entering Intrinsic capacity and cognition as continuous predictors. Both predictors standardised within the robust subgroup, so the hazard ratio is expressed per SD among robust participants.	

## Data Availability

LASI-DAD data are available to researchers on application through the Gateway to Global Aging Data (https://g2aging.org) and the LASI-DAD study (https://lasi-dad.org).

## Key summary points

- **Aim:** To determine whether intrinsic capacity adds to the physical frailty phenotype in predicting mortality in older adults, which IC domain carries that information, and whether reliance on the physical phenotype alone leaves a high-risk group unidentified.
- **Findings:** In 3,260 older Indians, intrinsic capacity and the physical phenotype were complementary predictors of mortality, and the information IC added beyond the physical phenotype was specifically cognitive; physically robust but low-IC participants died at approximately twice the rate of robust, high-IC participants — comparable to those meeting physical frailty criteria.
- **Message:** Pairing a brief cognitive assessment with physical frailty screening identifies high-risk older adults who would otherwise be reassured by a physical screen.
